# Co-Delivery of Berberine Chloride and Tariquidar in Nanoliposomes Enhanced Intracellular Berberine Chloride in a Doxorubicin-Resistant K562 Cell Line Due to P-gp Overexpression

**DOI:** 10.3390/pharmaceutics13030306

**Published:** 2021-02-26

**Authors:** Giulia Vanti, Marcella Coronnello, Daniele Bani, Antonella Mannini, Maria Camilla Bergonzi, Anna Rita Bilia

**Affiliations:** 1 Department of Chemistry “Ugo Schiff”, University of Florence, via Ugo Schiff 6, 50019 Sesto Fiorentino, Italy; giulia.vanti@unifi.it (G.V.); mc.bergonzi@unifi.it (M.C.B.); 2 Department of Health Sciences, Clinical Pharmacology and Oncology Section, University of Florence, Viale Pieraccini 6, 50139 Firenze, Italy; marcella.coronnello@unifi.it; 3 Research Unit of Histology & Embryology, Department of Experimental and Clinical Medicine, University of Florence, Viale Pieraccini 6, 50139 Firenze, Italy; daniele.bani@unifi.it; 4 Department of Experimental and Clinical Medicine, Section of Internal Medicine, University of Florence, Viale GB Morgagni 50, 50134 Firenze, Italy; antonella.mannini@unifi.it

**Keywords:** MDR, berberine chloride, tariquidar, nanoliposomes, endocytosis, uptake

## Abstract

The MDR phenomenon has become a major obstacle in the treatment of cancers, and among the strategies to reverse it, the inhibition of P-gp function and expression is essential to increase for effective anticancer drugs. In the present paper, the co-delivery of berberine chloride and tariquidar loaded nanoliposomes was investigated with the aim of enhancing solubility and improving desired effects for the antineoplastic drug and the P-gp inhibitor. Developed nanoliposomes were loaded with the electron-dense enzyme horseradish peroxidase, and analyzed by TEM to investigate their ability to enter in both K562 and K562/DOXO cell lines. Receptor-mediated endocytosis was evidenced for both cell lines. Nanoliposomes were loaded with tariquidar, berberine chloride, or both, maintaining chemical and physical characteristics—i.e., size, homogeneity, and encapsulation efficiency—and high suitability for parenteral administration. Tariquidar was able to reverse the MDR in the K562/DOXO cell line. Tariquidar- and berberine chloride-loaded nanoliposomes showed a significant increase of berberine chloride accumulation in tumor cells, which could be correlated with resensitization of the resistant cells to the antitumor agent. These results suggest that the co-delivery of the P-gp inhibitor, tariquidar, and the cytotoxicity inducer, berberine chloride, looks like a promising approach to overcome the MDR.

## 1. Introduction

Drug resistance is a well-known phenomenon and represents the cause of the non-responsiveness of some diseases to drug treatments. The occurrence was firstly described in bacteria resistant to antimicrobial drugs [1], but similar mechanisms have been found to take place in other diseases, including cancer. Nowadays, it is widely known the multidrug resistance (MDR), a cross-resistance to a broad variety of anticancer drugs unrelated in structure and activity, which produces chemotherapy failure and tumor progression. One of the most studied mechanisms of MDR is the overexpression of drug efflux pumps belonging to the adenosine triphosphate (ATP) binding cassette (ABC) transporters family, characterized by a broad and overlapping spectrum of substrates. The main ABC transporters clinically associated with the development of MDR are P-glycoprotein (P-gp/ABCB1), MDR related proteins (MRPs/ABCCs), and breast cancer resistance protein (BCRP/ABCG2) [2]. Nowadays, the MDR phenomenon represents a major obstacle in the treatment of cancer diseases, and among the strategies to reverse the MDR phenotype, the inhibition of P-gp function and expression is essential to discover fruitful anticancer drugs [3]. This hindrance can be overcome by the co-administration of antitumor drugs with substances able to inhibit MDR transporters such as P-gp. Such agents are known as chemosensitizers, as they can resensitize cancer cells to antineoplastic drugs, increasing the drug intracellular concentration by the P-gp efflux pump inhibition [4]. However, although several compounds show an inhibitor effect on the function of P-gp, clinical trials have revealed that most of these chemosensitizers are toxic, with limited benefits to cancer patients. Therefore, new strategic approaches are needed to increase the efficacy of these inhibitors and decrease their toxicity [5]. The co-encapsulation within delivery systems has been experimented to improve pharmacokinetic and pharmacodynamic features of P-gp inhibitors [6]. Among the nanocarriers, liposomes represent the most versatile drug delivery systems in terms of drug loading, biocompatibility, nontoxic, and non-immunogenic properties, and with an excellent biodegradability [7]. Hence, liposomes can enhance drug solubility, giving a sustained release system, providing targeted drug delivery, reducing the toxic effect of drugs, protecting against drug degradation, and the surrounding environment [8,9]. In clinical practice, liposomal formulations of important and effective drugs against both neoplastic and other diseases have been employed for many years, e.g., Doxil^®^, Ambisome^®^, DepoDur, Myocet, DaunoXome, DepoCyte, and Lipoplatin, are the most representative among others [10].

**Figure 1:**
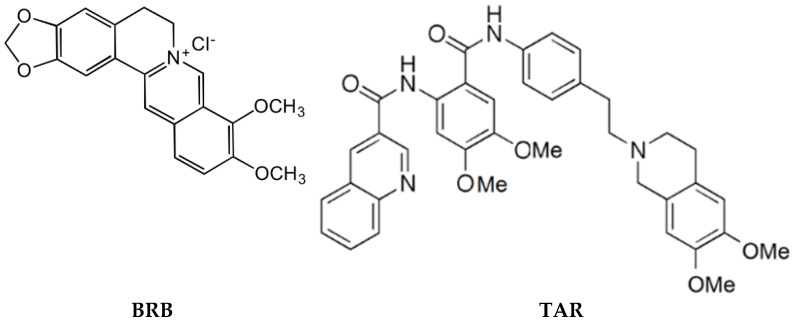
Chemical structures of berberine chloride and tariquidar.

In the present paper, the co-encapsulation of berberine chloride (BRB) and tariquidar (TAR) of nanoliposomes was investigated with the aim of enhancing solubility, and improving desired effects, both of the antineoplastic drug and the P-gp inhibitor. BRB is the more commonly available salt form of a natural quaternary ammonium isoquinoline alkaloid (Figure 1).

BRB, originally used as a broad-spectrum antibacterial drug, has a wide range of pharmacological activities, including inhibitory effects on a variety of tumors. BRB affects the molecular mechanisms that cause tumors through various signaling pathways, and it diminishes the expression of genes encoding cytokines involved in inflammatory pathways (e.g., tumor necrosis factor-α (TNF-α), interleukins), prostaglandins, cyclooxygenase−2 (COX−2), and inducible nitric oxide synthase (iNOS) via interfering with AMP-activated protein kinase [11,12,13]. Despite these interesting antineoplastic activities, BRB is a substrate of P-gp [14], and eliminated from the cells, resulting in reduced antitumor efficacy. Furthermore, due to its poor aqueous solubility, BRB shows a very low oral bioavailability (<1%) [14,15]. Studies in humans showed that BRB efflux is due to multidrug resistance protein 1 and multidrug resistance-associated protein 1, reducing accumulation of BRB in cells, but it is also well-known BRB can also bind strongly to human serum albumin, which makes it difficult to achieve effective concentrations. Metabolic studies of BRB in animals suggest that BRB is metabolized rapidly and that the liver is the main metabolic site. It was shown that only 4.93% and 0.5% of an i.v. dose of 2 mg/kg BRB was eliminated from the urine and bile, respectively [16,17]. Hence the need for an adequate drug delivery system to increase BRB bioavailability. TAR (XR9576, Figure 1) is a third-generation synthetic potent P-gp inhibitor, whose potential to overcome P-gp mediated MDR of tumors has been investigated in human phase I/II clinical trials. However, further clinical development of TAR as a MDR reversal agent halted due to the scarce efficacy and side effects when used in combination with cytotoxic anticancer drugs [18]. In addition, in the present study D-α-Tocopheryl polyethylene glycol 1000 succinate (TPGS) was also investigated as a possible P-gp inhibitor and compared with TAR to select the best MDR reversal agent [19]. The first step of the study was characterized by the optimization of composition of the nanoliposome bilayer, based on the key properties—such as particle size, polydispersity, ζ-potential, and morphology. Encapsulation efficiency (EE%) and recovery (R%) of drugs were also evaluated. Transmission electron microscopy analysis (TEM) in both the parental human leukemia cell line K562 and the doxorubicin (DOXO) resistant counterpart for P-gp overexpression, K562/DOXO, was used to investigate the endocytosis properties of the developed nanoliposomes loaded with horseradish peroxidase (an electron-dense enzyme). Possible changes in the potency of TAR loaded in nanoliposomes was evaluated using DOXO cytotoxicity in both cell lines study. Finally, BRB- plus TAR-loaded nanoliposomes were investigated for the ability to enhance BRB uptake in K562/DOXO cell lines. Due to its fluorescent properties, the BRB is a suitable probe for evaluating directly the uptake by flow cytometry [20].

## 2. Materials and Methods

### 2.1. Chemicals

Phosphotungist acid (PTA) was purchased from Electron Microscopy Sciences (Hatfield, PA, USA). Berberine chloride (BRB, C_20_H_18_ClNO_4_, PM: 371.81 g/mol), sodium chloride, cholesterol (C_27_H_46_O, 95%, PM: 386.65 g/mol), sodium hydroxide (NaOH, PM: 40 g/mol), Phosphate buffer (PBS 0,01 M, pH 7.4 (NaCl 29 mM, KCl 2.5 mM, Na_2_HPO_4_⸱7H_2_O 7.4 mM, KH_2_PO_4_ 1.3 mM), D-α-Tocopherol polyethylene glycol 1000 succinate (TPGS), acetonitrile and methanol HPLC grade, dichloromethane, dimethyl sulfoxide (DMSO), ethanol, aqueous glutaraldehyde aqueous solution (25% v/v), trypan blue, 3-(4,5-dimethylthiazol−2-yl)−2,5-diphenyltetrazolium bromide were from Sigma-Aldrich (Milan, Italy). Phospholipon 90G (P90G: phosphatidylcholine 90%, PM: 775 g/mol), and Phospholipon 100H (P100H: hydrogenated phosphatidylcholine 100%), were purchased from Lipoid AG (Cologne, Germany) with the support of its Italian agent AVG srl. Tariquidar (TAR, C38H38N4O6, 98.57%, PM: 646.73g/mol) was from MedChemExpress (Moltport, Sweeden) bidistilled water purified by MILLIQ-plus from Millipore (Milford, MA, USA); doxorubicin (DOXO) was from TEVA Pharmaceutical industries (Milan, Italy); FITC Annexin/Dead cell Apoptosis Kit with FITC annexin V and propidium iodide (PI) for Flow Cytometry; culture medium RPMI 1640, fetal bovine serum (FBS), l-glutamine, penicillin G and Streptomycin were from Gibco^®^, European Division (San Giuliano Milanese, Italy); Peroxidase Type VI was from *Armoracia rusticana* L. (horseradish, PM 44,000 g/mol, Sigma-Aldrich, Milan, Italy).

### 2.2. Cell Cultures

K562 human erythroleukemia parental cell line (ATCC, Rockville, MD, USA) were used [21]. Cells grew in RPMI 1640 culture medium added with penicillin G 100 U/mL, streptomycin 100 mg/mL, L-glutamine 2 mM and 10% fetal bovine serum (FBS), at 37 °C in a humidified atmosphere at 5% of CO_2_. The culture was maintained by two weekly passages in complete fresh medium.

The DOXO-resistant K562 clone (K562/DOXO), was obtained by exposure of the parental K562 cells to increasing drug concentrations up to 400 nM DOXO, using the same culture medium of the parental line, and characterized by expression of a unique membrane P-gp (180 kD molecular weight). To maintain the P-gp overexpression, the K562/DOXO cells were monthly exposed to 400 nM DOXO for 72 h. After DOXO treatment, the cultures were washed by centrifugation (5 min at 1200 rpm), resuspended and incubated in the drug-free growth medium, at 37 °C in a humidified atmosphere at 5% of CO_2_. This maintenance method represents the maintenance routine of the doxo-resistant cell line; these cells are used after 3 weeks of culture in drug-free medium.

### 2.3. HPLC-DAD Analytical Method for the Evaluation of BRB, TPGS, and TAR

Evaluation of BRB, TAR, and TPGS was obtained by the 1200 high performance liquid chromatography apparatus (HPLC) equipped with a diode array detector (DAD), (Agilent Technologies Italia Spa, Rome, Italy). The DAD wavelengths for TAR, TPGS, and BRB were 240, 284, and 346 nm, respectively. Chromatography was performed at 27 °C, using a gradient elution method with (A) acetonitrile and (B) water at pH 3.2 (by formic acid), as mobile phases and a reverse-phase column Luna-C18 (250 × 4.6 mm, 5 μm particle size). The flow rate was 1 mL/min for 35 min. The analytical method was 0.1–3 min 85% (B), 3–9 min 85–70% (B), 9–10 min 70% (B), 10–17 min 70–50% (B), 17–25 min 50–1% (B), 25–35 min 1–85% (B). Acetonitrile was selected as the low UV cut off-mobile phase, in order to avoid the interference with the TAR signal. The external standard method was used for the quantitative determination of the investigated drugs during the whole set of experiments. BRB calibration curve was prepared using a stock solution of BRB in HPLC grade MeOH at a concentration of 0.54 mg/mL, which was subsequently diluted 2, 5, 10, 20, 50, and 100 times with HPLC grade MeOH. The linearity of the calibration curve obtained, expressed by the R^2^ value, resulted 0.99998. TAR calibration curve was set using a stock solution of TAR at a concentration of 0.253 mg/mL in a mixture of CH_2_Cl_2_ and MeOH with a 25:75 ratio. Subsequently, appropriate dilutions, namely 2, 5, 10, and 100 times with HPLC grade MeOH, were prepared. R^2^ value resulted in 0.99972. A stock solution of TPGS in HPLC grade MeOH had a concentration of 0.320 mg/mL, subsequently diluted 2, 5, 20, 50, and 100 times with HPLC grade MeOH. R^2^ value resulted in 0.99981.

### 2.4. Preparation and Optimization of Nanoliposomes

The nanoliposomes were prepared according to the thin layer evaporation method as previously reported [22]. Two phospholipids, namely natural P90G and P100H, and cholesterol were used for the preparation of the vesicles, testing different combinations and gravimetric ratios, as reported in Table 1. Briefly, the lipid components were weighed, solubilized in a 250 mL glass flask with CH_2_Cl_2_, and then, for the complete solubilization of the components, were sonicated for 1–2 min in the ultrasonic bath. The organic solvent was removed by a rotary evaporator for 30 min at 35 °C, and the obtained dried lipid film was hydrated with 10 mL of PBS, by stirring at 650 rpm for 30 min, at 35 °C (P90G) and 55 °C (P100H), temperatures higher than the phospholipid transition temperature. In order to optimize polydispersity index (PdI) and sizes of nanoliposome suspension, ultrasonication by SONOPULS Ultrasonic Homogenizer HD 2200 (Bandelin electronic GmbH & Co. KG; Berlin, German) and MS72 or KE76 probes was carried out for 5 min at 5 cycles and 50% power, in an ice bath. Subsequently, to remove possible metal particles released by the probe, the samples were centrifuged at 25 °C for 1 min at 2000 rpm.

#### 2.4.1. Formulation of Nanoliposomes Loaded with BRB (BRB-L)

The nanoliposomes loaded with BRB were prepared according to the previous method, by adding 0.7 mg/mL of BRB to the final liposomal suspension. Briefly, P90G, cholesterol, and BRB were weighed in different gravimetric ratios (Table 2) and they were solubilized in a 250 mL glass flask with a mixture of CH_2_Cl_2_ and MeOH, using the ultrasonic bath. Successively, the preparation method continued as previously reported, until the ultrasonication of lipid suspension in 10 mL PBS.

#### 2.4.2. Formulation of Nanoliposomes Loaded with P-gp Inhibitors (TAR-L and TPGS-L)

The liposomal formulations made of 660 mg of P90G plus 100 mg of Chol, and loaded with TAR or TPGS, were developed using the method reported in Section 2.4. Briefly, TAR or TPGS were added to the organic phase of CH_2_Cl_2_/MeOH at the final concentration of 0.647 mg/mL (1mM) and 7.6 mg/mL (13 mM), namely 10% w/w of the lipid component weight, respectively. The obtained formulations were optimized by performing 10 or 5-min sonication for TAR-L and TPGS-L, respectively, at 5 cycles and 50% power, using the homogenizer with MS72 probe.

#### 2.4.3. Formulation of Nanoliposomes Loaded with BRB Plus TAR (BRB/TAR-L)

The procedure of this formulation was the same reported previously for nanoliposomes loaded with BRB (Section 2.4.1) using the following lipid ratio: 660 mg of P90G and 100 mg of Chol. BRB plus TAR were added in the organic solvent to reach a final concentration of 0.7 mg/mL and 0.647 mg/mL, respectively. The optimization was carried out using a 5 min sonication at 5 cycles (50% power, MS72 probe).

### 2.5. Physical Characterization and Morphological Study of Nanoliposomes by Transmission Electron Microscope (TEM)

During aa optimization procedures, nanoliposome characterization has been performed by evaluating size, homogeneity, and possible aggregation state, with the aid of dynamic and electrophoretic light scattering (DLS and ELS, Zetasizer Nanoseries ZS90) by Malvern Instruments (Worcestershire, UK), by using a scattering angle of 90 °C at 25 °C [23]. Autocorrelation functions were analyzed by the cumulant method in order to obtain the average hydrodynamic diameter (AHD, nm), the size distribution expressed as PdI (polydispersity index, dimensionless measurement) and ζ-potential (mV), using the software provided by Malvern. Scattering measurements, in triplicate, were performed on samples, diluted 50/100-fold in ultrapure water. Nanoliposome morphology was investigated for vesicle dispersion, dimension, and deformability by TEM (CM12 TEM, Philips, The Netherlands) equipped with an Olympus Megaview G2 camera, applying an 80 kV accelerating voltage. For this purpose, a drop of the diluted sample, released on a carbon film copper grid, was dried by desiccation, counterstained with 1% (*w*/*v*) of phosphotungstic acid solution and examined at different magnifications [24].

### 2.6. Chemical Characterization of Nanoliposomes: Encapsulation Efficiency (EE) and Total Recovery (R)

For each liposomal formulation, the encapsulation efficiency (EE%) and the total recovery (R%) of BRB and TAR were calculated. EE% is expressed as the percentage of the ratio of entrapped and weighted drug (Equation (1))

EE% = encapsulated drug/weighted drug × 100(1)

In order to evaluate the entrapped drug, nanoliposomes were purified from free BRB and free TAR or TPGS, by dialysis bag method [25], using Spectra/Por^®^ regenerated cellulose membranes (12–14 KDa molecular weight cut-off) (MWCO) (Repligen Europe B.V., Breda, The Netherlands). Briefly, the dialysis bag was stirred at 100 rpm, for 30 min, in ultrapure water (1 L), at 25 °C. Then, to dissolve and disrupt the nanoliposome membranes, the purified samples were diluted in methanol and the encapsulated substances were released. The process was enhanced by immersing the diluted samples in ultrasonication bath for 30 min. All samples were centrifuged two times at 14,000 rpm for 10 min and analyzed by HPLC-DAD. Recovery (R%), defined as the percentage of total recovered drug after the preparation procedure in relation to the weighed drug, was measured using the same procedure described for the EE% without the dialysis purification step, and applying the following Equation (2)

R% = total recovered drug/weighted drug × 100(2)

### 2.7. TEM Analysis of K562 and K562/DOXO Cells Incubated with Peroxidase-Loaded Nanoliposomes

In order to better understand the mechanism of interaction of the nanoliposomes with the K562 cell membrane, nanoliposomes loaded with horseradish peroxidase were prepared, fully characterized, and incubated with K562 or K562/DOXO cells. Since nanoliposome horseradish peroxidase works as a probe, the fine morphological interactions of nanoliposomes may be followed by TEM evaluation and analyzed with DLS. Briefly, a nanoliposomal formulation loaded with a solution of peroxidase in PBS (1 mg/mL) was prepared using TLE method as reported previously. A 10 mL measure of this solution was used to hydrate the lipid film by a mechanical stirring at 37 °C for 30 min at 1300 rpm. The optimization of formulation was carried out by 5 min ultrasonication with 0.5 **s** intervals, without cycles, at a maximum intensity of 50%. K562 or K562/DOXO cells were incubated with peroxidase-loaded nanoliposomes, for 15 and 120 min, respectively. Peroxidase-loaded nanoliposomes, K562 or K562/DOXO cells, submitted at the same protocol, were centrifuged, fixed in Karnovsky liquid (2% paraformaldehyde and 4% glutaraldehyde in 0.2 M cacodylate buffer, pH 7.4), post-fixed in 1% OsO4 in 0.1 M phosphate buffer, pH 7.4, dehydrated in a series of acetone with increasing titer, passed briefly in propylene oxide and finally included in epoxy resin (Epon812). The analysis was conducted on ultra-fine sections with a thickness of about 80 nm, obtained with an Ultrotome III ultramicrotome, contrasted with uranyl acetate and alkaline bismuth sub-nitrate. The observation was made at TEM at an accelerated voltage of 80 kV. The micrographs were taken with a digital camera connected to the microscope.

### 2.8. In Vitro BRB Release from BRB-L or BRB/TAR-L

The release of BRB from BRB-L and BRB/TAR-L was investigated over a period of 2 h, in presence of the culture medium (RPMI medium 1640 with 10% FBS, fetal bovine serum), to mimic the cell incubation conditions. A comparison of the release properties of the formulation with an aqueous BRB solution at the same concentrations was carried out. The experiment was performed on dialyzed nanoliposomes (according to the procedure described in Section 2.6), using the dialysis bag method and the Spectra/Por^®^ regenerated cellulose membranes of 12–14 KDa MWCO by Repligen Europe B.V. (Breda, The Netherlands) [26]. BRB-loaded nanoliposomes and BRB aqueous solution were diluted (DF = 10) in the culture medium, and an exact volume (3 mL) of the suspension/solution was transferred into the dialysis bag. The experiment was carried out in 100 mL of PBS (pH 7.4), selected as medium and maintained at the constant temperature of 37 °C by a magnetic stirrer (100 rpm) with a heating plate. At specified time points (15, 30, 45, 60, 75, 90, 105, 120, 180, 240, and 300) min the medium was taken for the analysis and replaced by equal volumes of fresh buffer, in order to maintain the sink conditions. Finally, all samples were centrifuged at 14,000 rpm for 10 min and analyzed by HPLC-DAD. The amount of released BRB was expressed as the percentage of the drug released in the medium divided by the drug present inside the bag. The amount of BRB released during the study was expressed by the following formula (Equation (3))

% release = (released drug)/(drug inside the bag) × 100(3)

### 2.9. Stability Studies of BRB-L and BRB/TAR-L in Cell Culture Medium

The stability of developed BRB-L and BRB/TAR-L within the culture medium (RPMI medium 1640 with 10% FBS) was evaluated. Aliquots of the dialyzed formulations were diluted 10 times in the culture medium (as in the in vitro tests) and incubated for 2 h at 37 °C, using the PST−60HL−4 Thermo-Shaker (Biosan). After the incubation time, physical (size, PdI, ζ-potential), and chemical (EE% and R%) parameters of nanoliposomes were determined by DLS/ELS and HPLC-DAD.

### 2.10. Cytotoxicity of DOXO in the Absence and in the Presence of Free or Liposomal TAR or TPGS

Cell death by apoptosis was determined after treatment of the K562/DOXO cell line with DOXO in the absence and in the presence of free or liposomal TAR and TPGS. The test was also carried out using empty liposomes. The solution of TAR or TPGS in DMSO was prepared at a concentration 10 times higher than that present in the nanoliposome formulations, and diluted in the cell suspension with an DF = 100, in order to reduce the percentage of DMSO to 1% *v*/*v*, avoiding cell toxicity. Similarly, TAR-loaded liposomes were diluted in the cell suspension with a DF = 10. DOXO in DMSO was added in order to obtain a final concentration equal to 10 μm. The final concentrations of TAR and TPGS in the cell wells were 10 μm and 13.2 μm, respectively. The samples were incubated for 1 h at 37 °C in a humidified atmosphere with 5% CO_2_. An aliquot of samples (3 mL) from each well were collected after 15, 30, 45, and 60 min, washed twice with PBS, and resuspended in complete medium and incubated for 24 h at 37 °C in a humidified atmosphere with 5% CO_2_. At the end of the incubation, the cell samples were counted and prepared for the detection of apoptotic and/or necrotic cell percentage, using a cytometer. A kit containing annexin V conjugated with fluorescein isothiocyanate (FITC) and a solution of propidium iodide (PI) was used. The cells are centrifuged at 1200 rpm for 5 min, and the cell pellets (approximately 1 × 10^6^ cells) were resuspended in 100 µL of buffer A (solution of annexin-binding in deionized water) and 5 µL of FITC-Annexin plus 1 µL of the PI 1X (100 µg/mL buffer A) solution added to the 100 µL of cell suspension. Cells were incubated at room temperature for 15 min. After 400 µL of buffer A were added and samples were shaken gently, leaving them in ice and in the dark until the time of analysis on the cytometer. With this kit the viable cells do not acquire any fluorescence, the necrotic cells stain red as they have the damaged membrane, the cells in the early phase of apoptosis are colored green and the cells in the late phase of apoptosis stain both red and green. The different cell populations can then be distinguished using a flow cytometer with an excitation wavelength of 488 nm and accessorized with filters with an emission wavelength of 530 nm for the acquisition of apoptotic cells stained green with FITC and of a 620 nm emission filter for the acquisition of necrotic cells stained red with the PI. Cells stained in both green and red represent cells in late apoptosis.

### 2.11. Uptake Studies by Flow Cytometry

BRB, being a fluorescent molecule, is a suitable probe to measure cell uptake and the functionality of the P-gp pump in K562/DOXO cell line, resistant to doxorubicin due to overexpression of this pump. Flow cytometry was used to evaluate cellular uptake of fluorescent BRB in human parental and DOXO-resistant K562 cell line. A cell density of approximately 8 × 10^5^ cells/mL, in the exponential growth phase, was used. Samples were prepared by diluting the formulations in the cell suspension with DF = 10. The solution of BRB and TAR in DMSO were prepared at a concentration 10 times higher than the concentration present in the formulations, and diluted in the cell suspension with an DF = 100 in order to reduce the percentage of DMSO to 1% *v*/*v* and to avoid cell toxicity. The final concentration of TAR in the cell wells was 10 μM. The P-gp inhibitor was added 15 min before BRB. Preliminarily, two incubation times with the nanoliposomes were used, namely 2 and 4 h (data not shown). Since the results showed a similar uptake, therefore a single exposure time was chosen for the subsequent experiments—i.e., 2 h at 37 °C in a humidified atmosphere with 5% CO_2_. Aliquots of each sample suspension were used to establish uptake of the BRB. Therefore, in order to evaluate the uptake, 1 mL of each sample was washed twice with cold PBS, by centrifugation at 1200 rpm for 5 min at 4 °C to remove non-internalized BRB. The pellet of each sample was then resuspended in 1 mL of cold PBS and kept on ice, in the dark, until read at the cytometer. The fluorescence of BRB (λ_ex_ 488nm/λ_em_ nm, 530 ± 30 nm) was explored to measure the degree of cell accumulation. All samples were acquired by a FACS Canto flow cytometer (Becton Dickinson) and for each sample 20,000 events were acquired, analyzed by FCS 6 Express software (De Novo Software, Glendale, CA, USA), and transformed into fluorescence histograms. The fluorescence ratio (RF) of treated samples and the control sample (empty liposomes) is evaluated.

### 2.12. Statistical Analysis

All experiments were independently carried out at least three times. All results represented as means ± SD and statistical analysis was performed using the one-way ANOVA test and Bonferroni’s multiple comparison test (GraphPad Prism software, Inc., San Diego, CA, USA). *p* < 0.05 was considered significant.

## 3. Results

### 3.1. Formulation of Nanoliposomes and Characterization

Different combinations and gravimetric ratios of P90G or P100H plus Chol were tested (Table 1) in order to select, by DLS analysis, the best formulations to load BRB.

The hydration of the lipid film by using glass beads led to multilamellar vesicles with dimensions ranging from 800 to 1000 nm, which were optimized by ultrasonication, as reported in the experimental part. P90G plus Chol was found to be the best combination for the preparation of suitable nanoliposomes for parenteral administration in terms of size (less than 200 nm) and homogeneity (PdI less than 0.3).

All the nanovesicles based on P90G plus Chol had good PdI (0.20–0.25), but the nanoliposomes having P90G/Chol concentration ratio of 65:30 mg/mL were larger than 200 nm and not suitable for the parenteral administration. The liposomal formulation 66:10 (P90G:Chol) was finally selected on the basis of the physical characteristics and BRB loading ability. Increasing amounts of BRB were loaded in the nanoliposomes to reach a maximum of 0.7 mg/mL using the different P90G/Chol ratios, in order to maximize the optimal size and PdI for the parenteral route, as well as the highest encapsulation of BRB. Table 3 describes the physical and chemical characteristics of the investigated BRB nanovesicles (BRB-L).

Nanoliposomes based on P90G/Chol with a concentration ratio of 66:10 (mg/mL) showed highest encapsulation efficiency of BRB (ca. 64%). These nanoliposomes had also an optimum ζ-potential value (−20.3 ± 2.5 mV) to protect nanoliposomes from aggregation and precipitation.

These nanoliposomes were selected to load also Pg-p inhibitors—i.e., TAR (TAR-L) and TPGS (TPGS-L)—and to formulate BRB plus TAR-loaded nanoliposomes (BRB/TAR-L). TAR concentration was 0.647 mg/mL (1 mM) in both liposomes, whereas BRB concentration was 0.7 mg/mL. The physical characterization (*n* = 3) was carried out by DLS. BRB/TAR-L evidenced sizes of 137.9 ± 11.7 nm, and PdI of 0.25 ± 0.03. In addition, R% and EE% (*n* = 3) of BRB and TAR, determined by HPLC-DAD, were found to be 99.43 ± 2.42% and 57.01 ± 11.1% for BRB, and 85.64 ± 4.24% and 78.76 ± 3.03% for TAR. TAR-L had similar R% and EE%, average sizes of 141.3 ± 0.4 nm and PdI of 0.184 ± 0.004. TPGS-L had an EE% near to 100% and dimensions and PdI values similar to the unloaded nanovesicles.

**Figure 2:**
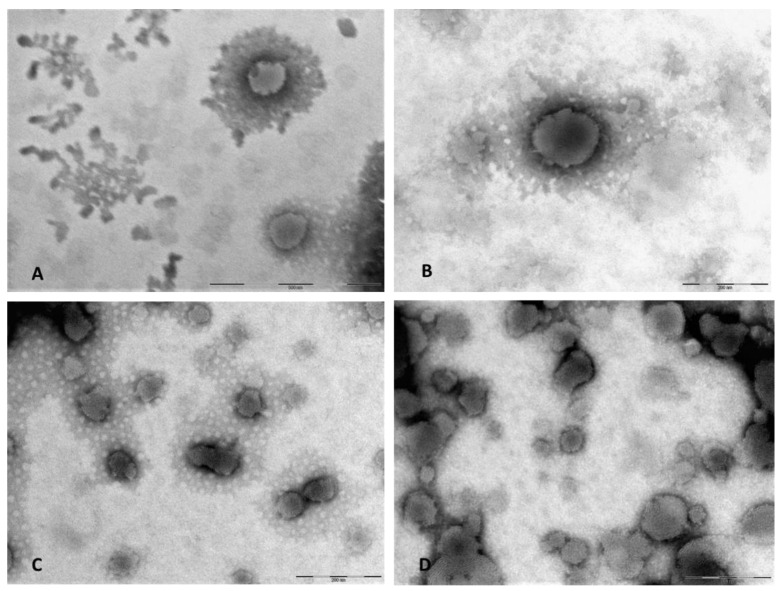
TEM pictures of BRB-L before (**A**) and after dialysis process (**B**), and of BRB/TAR-L before (**C**) and after dialysis process (**D**).

Finally, the morphological characterization of the nanoliposomal was carried out by TEM. The microscopic analysis highlighted the vesicular and spherical structure of all the developed nanoliposomes, confirming the sizes and the homogeneity of the samples, in agreement to the DLS data. In particular, TEM analysis of BRB-L and BRB/TAR-L, performed before and after the dialysis step, evidenced that both formulations were not affected by this purification process (Figure 2A–D).

### 3.2. Stability Studies of Nanoliposomes in Cell Culture Medium

Physical and chemical stability of the dialyzed BRB-L and BRB/TAR-L was evaluated after incubation for 2 h at 37 ± 2 °C in the culture medium (RPMI medium 1640 at 10% FBS). At the end of the incubation time, each sample was analyzed by DLS and HPLC-DAD (Table 4). Both nanoliposomes were physically stable (*n* = 3) over time. BRB-L showed a slight increase (less than 4%) for both size and PdI after two hours of incubation. BRB/TAR-L, after two hours of incubation, showed a small decrease of the hydrodynamic diameter (from 137.6 nm to 135.3 nm), while the PdI reached the value of 0.332 (Table 4).

In parallel, the amounts of BRB and TAR were monitored by HPLC-DAD (Table 4). Being nanoliposomes purified before testing, only EE% was determined after incubation. EE% was decreased a little for either BRB or TAR, in both nanovesicles.

### 3.3. BRB In Vitro Release from Nanoliposomes

**Figure 3:**
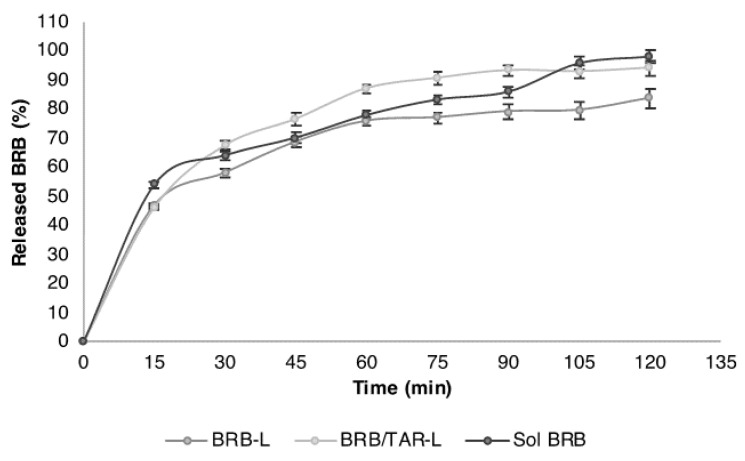
BRB release over time from dialyzed BRB-L and BRB/TAR-L, compared with BRB solution (BRB-SOL).

Figure 3 represents the release profiles of BRB from BRB-L and BRB/TAR-L, compared to the release from BRB solution (Sol BRB), obtained using 100 mL of PBS at pH 7.4 as release medium, at a constant temperature of 37 °C, for 2 h, and applying a magnetic stirring of 100 rpm. Before the test, BRB-L and BRB/TAR-L were dialyzed and diluted in the cell culture medium, in order to simulate the experimental conditions of the in vitro test. BRB release from both nanoliposomes was rapid. After 15 min, about 50% of BRB was recovered in the PBS external medium for both formulations. However, after 60 min the release became slower and more constant, reaching a BRB release of about 85% for BRB-L and approximately 95% for BRB/TAR-L, after 120 min. Accordingly, TAR did not greatly influence the release of BRB from the nanoliposomes.

### 3.4. Interaction of Horseradish Peroxidase-Loaded Nanoliposome with Cell Membranes of K562 and K562/DOXO Cell Lines by TEM

**Figure 4:**
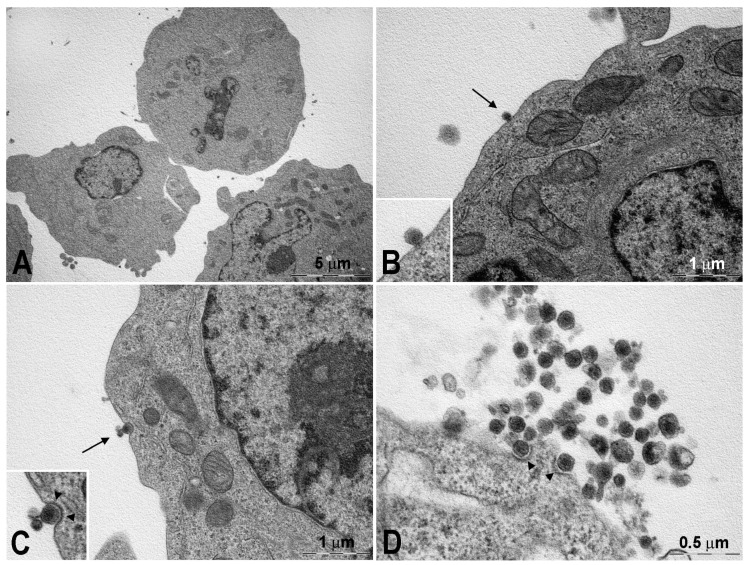
K562 cells after 15-min incubated with peroxidase-containing liposomes. (**A**) Cells still show a normal ultrastructure and one of them (top) appears in mitosis. (**B**) A liposome, in the form of a membrane-bound particle with electron-dense content, is attached to the plasma membrane (arrow and insert); to note that mitochondria show a normal appearance. (**C**) A liposome undergoing internalization by a coated pit (arrow and insert): the typical clathrin coating (arrowheads) is suggestive for a mechanism of receptor-mediated endocytosis. (**D**) A group of liposomes in proximity of a cell’s surface: some of them appear in the course of receptor-mediated endocytosis (arrowheads).

**Figure 5:**
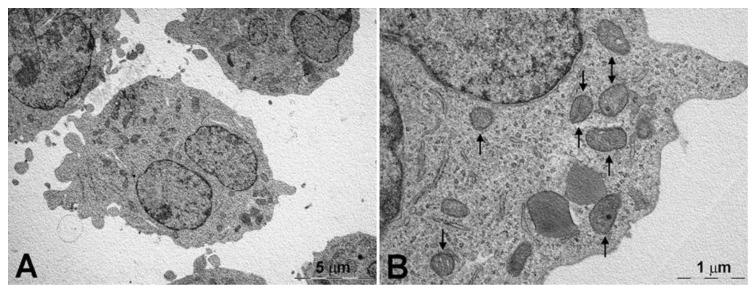
Control K562 cells. (**A**) The cells show a normal ultrastructure. (**B**) Numerous mitochondria (arrows) rich in cristae and with dense inner matrix-as usual in normally functioning organelles-can be seen in the cytoplasm.

**Figure 6:**
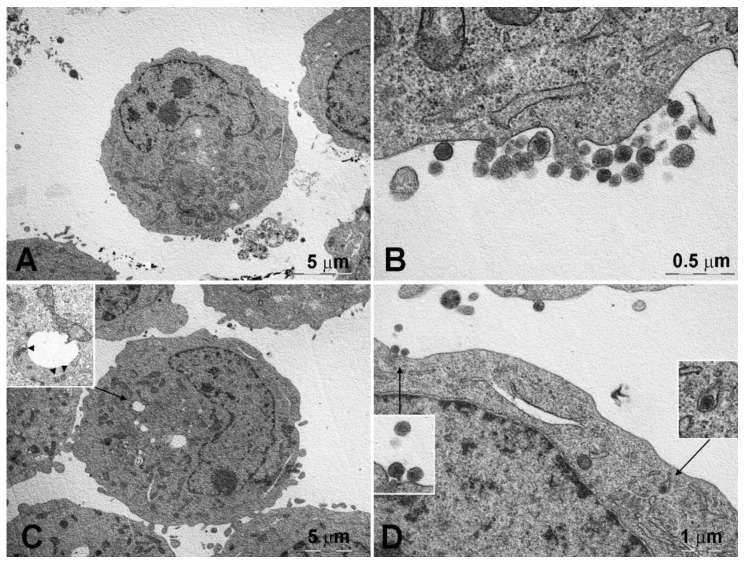
K562/DOXO cells after 15-min incubation with peroxidase-containing liposomes. (**A**) The cells show a normal ultrastructure and one of them (center) shows a cluster of membrane-bound, electron-dense particles, identified as liposomes, opposed to the plasma membrane. (**B**) Detail of the liposomes shown in (**A**), some of which are attached to the plasma membrane; mitochondria still show a normal appearance. (**C**) Cells showing moderate cytoplasmic vacuolation (see insert) and electron-dense particles corresponding to liposomes (arrowheads). (**D**) Detail of the cell in (**C**).

**Figure 7:**
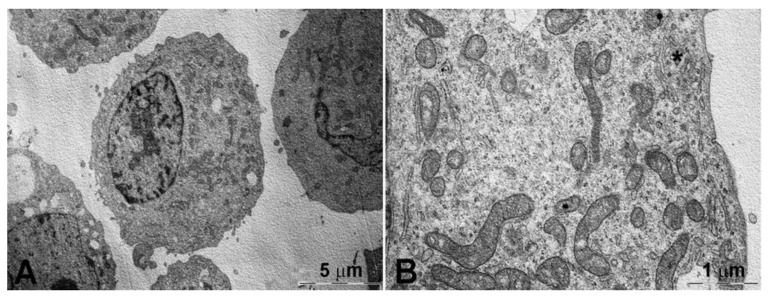
Control K562/DOXO cells. (**A**) Representative images of cells showing normal ultrastructure. (**B**) Rod-shaped mitochondria, with numerous cristae and dense inner matrix, typical features of normally functioning organelles, can be seen. * Golgi apparatus is indicated by the asterisk.

The interactions of developed nanoliposomes with cell membranes of K562 and K562/DOXO cell lines was studied by TEM (Figure 4, Figure 5, Figure 6 and Figure 7 and Figure S1 and Figure S2). Nanoliposomes were loaded with the electron dense enzyme horseradish peroxidase as reported in Section 2.7. DLS analysis of the developed nanoliposomes gave a size of 161.13 ± 19.33 nm and a PdI of 0.287 ± 0.051 (mean ± SD, *n* = 3). TEM highlighted the morphological interactions of horseradish peroxidase-loaded nanoliposomes with the cells, detecting their localization and structural shape within them.

The horseradish peroxidase-loaded nanoliposomes were incubated with the two cell lines and analyzed by TEM to evaluate their behavior after 15 (Figure 4 and Figure 6) and 120 min (Figure S1 and Figure S2) of exposure and compared with the controls (Figure 5 and Figure 7). After 15 min, the K562 cells (Figure 4) were surrounded by several membrane-bound particles with electron-dense content, consistent with liposomes by their size and shape: these particles adhered to the plasma membrane and sometimes appeared to undergo endocytosis by clathrin-coated pits and vesicles, suggesting the involvement of a mechanism of receptor-mediated endocytosis/phagocytosis.

After 15 min of incubation, the cells still showed a normal appearance, similar to that of the controls (Figure 5). After 2-h exposure to horseradish peroxidase-loaded nanoliposomes, most cells showed clear-cut signs of necrosis, with cytoplasmic vacuolization, swollen mitochondria with disruption of cristae, dispersed chromatin, multiple ruptures of the plasma membrane, and leakage of cytoplasm. Occasional liposome-like electron-dense particles were found close to the plasma membrane remnants and within cytoplasmic vacuoles (Figure S1).

The resistant K562/DOXO clone shows the same results at the investigated times (Figure 6, Figure 7, and Figure S2). Therefore, the horseradish peroxidase-loading nanoliposomes, once penetrated into the cell, cause irreversible damage of the cytomembranes, severe mitochondrial dysfunction, leading to cell death.

### 3.5. Cytotoxicity of DOXO in the K562/DOXO Cell Line Incubated with TAR, TAR-L, TPGS, and TPGS-L

**Figure 8:**
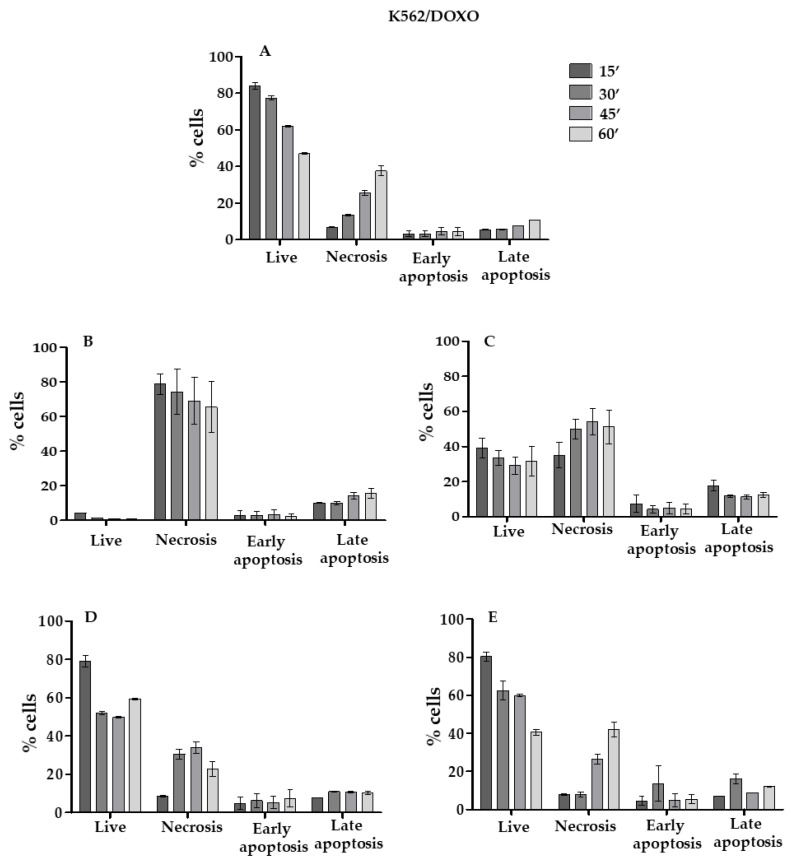
Effects of pump inhibitors (free or liposomal TAR and TPGS) on DOXO cytotoxicity in K562/DOXO cells. In particular, data are reported for DOXO (**A**), DOXO plus TAR (**B**), DOXO plus TAR-L(**C**), DOXO plus TPGS (**D**), and DOXO+TPGS-L (**E**). Cytotoxic effects are expressed as the percentage of live, necrotic, and apoptotic cells detected in the samples incubated with DOXO in the presence and in the absence of the different pump inhibitors free and formulated.

In order to select the most efficient pump inhibitor, the effect of DOXO, DOXO plus TAR, DOXO plus TPGS, DOXO plus TAR-L, or DOXO plus TPGS-L were studied using K562/DOXO cell line by the apoptosis assay, performed 24 h after the end of the incubation with DOXO, as reported in the experimental part (Figure 8). DOXO cytotoxicity was revealed after 60 min of DOXO incubation with K562/DOXO cells at a concentration of 10 μm.

A comparison of the results obtained in K562/DOXO cell line treated with DOXO and TAR solution (Figure 8B) or TAR-L (Figure 8C), highlighted that the necrotic cell percentage in the sample treated with free TAR is very high (approximately 80%) after 15 min treatment, remaining stable even in the other times.

The cell necrosis with TAR-L, in the first 15 min is low (approximately 50% of the free TAR), reaching, after 60 min, the 76% compared to that of free TAR.

As expected, TAR solution enables a better saturation of the P-gp pump rather than the TAR-L. However, interestingly, in the sample treated with the TAR-L, the percentages of both early and late apoptotic cells were higher when compared to sample treated with TAR solution. The TPGS was less active when compared with both free and formulated TAR. Its contribution to the cytotoxicity of DOXO is low with a very similar percentage of necrotic cells at 60 min to that of the sample treated with DOXO in the absence of the inhibitor. For this reason, TAR was selected as pump inhibitor for the co-delivery with BRB in nanoliposomes.

### 3.6. Uptake Studies

**Figure 9:**
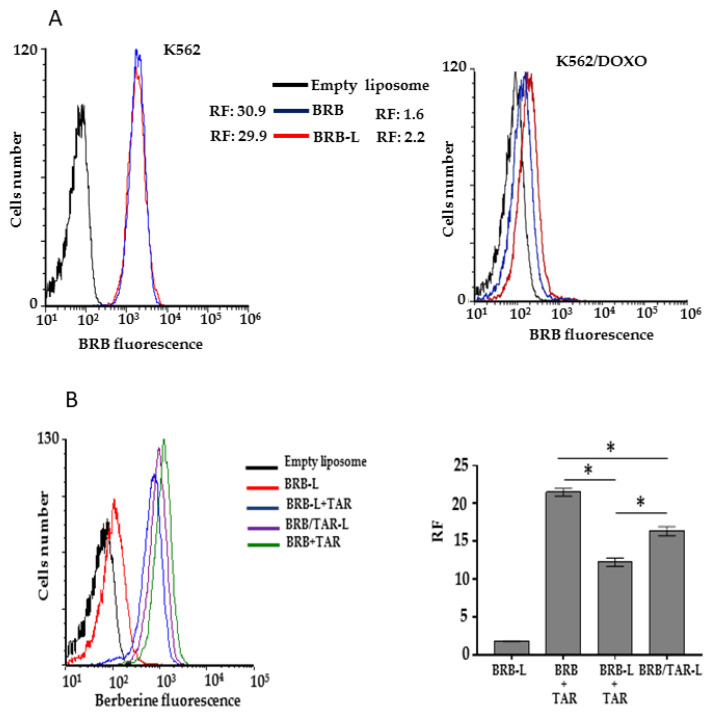
(**A**) uptake of BRB or BRB-L by K562 and K562/DOXO cells, after 2 h-incubation; (**B**) uptake of BRB/TAR, BRB-L/TAR, or BRB/TAR-L by K562/DOXO cells, after 2 h-incubation. RF = fluorescence ratios of sample treated and the control (empty liposome), * *p* < 0.05.

BRB, due to its intrinsic fluorescence, was used for the direct measurement of cell uptake, and the functionality of P-gp pump in the absence and in presence of TAR was investigated (Figure 9). The uptake of the BRB-loaded in nanoliposomes was tested by a FACS flow cytometer, using the fluorescence of the molecule directly. Specifically, K562 and K562/DOXO cell uptake of BRB-loaded in nanoliposomes was compared to a solution of free BRB (Figure 9A). The uptake of BRB in parental K562 cells after 2 h of incubation is not significantly different between the two BRB samples, the free molecule in solution or that encapsulated BRB in nanoliposomes. Therefore, the fluorescence ratios (RF) were comparable, overlapping the signals, with RF = 30.9 or RF = 29.9, when the cells were incubated in the presence, respectively, of the free BRB or BRB-L. K562 cells sensitive to DOXO did not show P-glycoprotein expression, and the use of any specific pump inhibitor is not necessary. On the contrary, K562/DOXO cells, having P-gp overexpression, showed a moderate increase in the accumulation of BRB-L.

Uptake in K562/DOXO cells was also evaluated after 2 h of incubation with BRB, BRB-L, and BRB plus TAR as free molecules in solution, BRB-L plus free TAR or BRB plus TAR both encapsulated in nanoliposomes (Figure 9B).

The BRB/TAR-L formulation gave a RF = 16, which represents the 76% of the value of the sample treated with BRB and TAR in solution (RF = 21) and is significantly different (*p* < 0.05) from the value of the BRB-L + TAR formulation (RF = 12); this feature makes it optimal for the simultaneous, safe, and effective release of BRB and TAR.

## 4. Discussion

In this paper, we investigated the simultaneous delivery of the anticancer drug, BRB, and a P-gp efflux pump modulator, TAR, using nanoliposomes to enhance BRB intracellular concentration. Untargeted liposomes loaded with antineoplastic drugs are the most successful nano-drug delivery systems translated into clinical applications and approved for their marketing, including for the treatment of hematological cancers. These nanocarriers minimize drug degradation and inactivation upon administration, as well as increase the drug’s bioavailability and the fraction of drug delivered within the pathological area, thus improving efficacy and/or minimizing drug toxicity. The developed nanoliposomal formulations encapsulating BRB plus TAR allowed us to increase the concentration of BRB inside the tumor cells, thus improving the potency. Nanoformulations were obtained by lipid hydration using different ratios of P100H or P90G plus Chol. The optimization of nanoliposome composition was carried out by fixing the concentration of P90G and P100H at values, which have been already optimized in previous studies, and solely modulating the content of cholesterol because of its key role in the rigidity of bilayers and as a consequence the loading of BRB. Accordingly, to the DLS measurements and BRB EE%, the selected nanocarriers were those based on P90G plus Chol, because P100H, the hydrogenated phosphatidylcholine, combined with Chol did not produced suitable nanoliposomes for parenteral administration.

Nanoliposomes made of P90G/Chol (66:10 mg/mL) had the best of BRB EE%, PDI and ζ-potential values. Furthermore, this formulation was clear and not viscous compared with the formulation having higher P90G/cholesterol concentration ratio (65:20 or 65:30 mg/mL). The same P90G/Chol ratio was maintained to formulate TAR-L, TPGS-L, and BRB/TAR-L.

As a first step of the study, the stability of the native and dialyzed nanoliposomes was assessed by TEM analysis because the purified liposomes were used in the tests. Dyalisis removal of unencapsulated BRB and TAR from nanoliposomes was revealed as a simple protocol to obtain purified liposomes without any variability of the size and morphology of vesicles and an efficient method to preserve the entrapped drug in the liposomes, as suggested by the HPLC-DAD analyses. In addition, the physical and chemical stability of nanoliposomes in the cell culture medium, RPMI medium 1640 with 10% FBS, were performed. Slight variance, less than 4%, for both the hydrodynamic diameter and PdI value were found. Overall, the tested formulations resulted physically and chemically stable under the experimental conditions, which are intended to mimic the in vitro studies within the two cell lines. Future studies will be oriented to the stability studies of the nanoformulations after storage in order to evidence the eventual need to lyophilize the nanoliposome in order to extend their shelf life.

Finally, release properties of the dialyzed nanoliposomes were also assessed using the same medium used for stability—i.e., RPMI medium 1640 with 10% FBS—evidencing good biopharmaceutical properties of nanoliposomes.

A key step of the present study was the evaluation of interaction of nanoliposome with cell membranes of K562 and K562/DOXO cell lines by TEM. The study was carried out using the enzyme horseradish peroxidase as electron dense material giving their localization and structural shape within the cells. TEM analysis evidenced the ability of the developed nanoliposomes to enter in both K562 and K562/DOXO cell lines by receptor-mediated endocytosis. This process was already evidenced after 15 min of incubation, but after 2 h exposure to horseradish peroxidase-loaded nanoliposomes, most cells showed clear-cut signs of necrosis, with cytoplasmic vacuolization, swollen mitochondria with disruption of cristae, dispersed chromatin, multiple ruptures of the plasma membrane, and leakage of cytoplasm. These findings suggested that peroxidase loaded in liposomes, likely due to the cytomembrane destabilization, is highly toxic for both cell lines, which are prompted to massive necrosis.

Furthermore, two P-gp inhibitors, TAR and TPGS both the free molecules and loaded in nanoliposomes were tested with DOXO in resistant cells, K562/DOXO, for their effects against overexpression of P-gp. TAR both in the free solution and encapsulated in liposomes was superior in overcoming DOXO resistance with respect to TPGS. In addition, from the cytotoxicity data it was hypothesized that the amount of Pg-p inhibitor available in the first instants of the experiment is greater with the inhibitor in solution, as nanoliposomes modify their release. In particular, it is worth noting that the toxicity of DOXO when treated with TAR-L is delayed compared to the toxicity observed in the sample treated with DOXO and TAR in solution.

Consequently, the advantage of the slower release is a reduction in cell death by necrosis. According to the literature, this is a process that heavily damages cells with leakage of the cell content into the surrounding environment and consequent cell death by in vitro necrosis and inflammatory processes in vivo [27]. This effect is strongly positive in clinical therapy and it is solely related to the nanodelivery system. Subsequently, the liposomes loaded with both BRB and TAR were optimized (BRB/TAR-L) and this formulation showed a significant increase in BRB uptake by the K562/DOXO cell line.

These results, together with the ability of TAR-L to significant resensitization of the resistant variant for DOXO, suggest that the co-delivery of the P-gp inhibitor, TAR, and the cytotoxicity inducer, BRB, looks like a promising approach to overcome the MDR.

The optimized co-administration system can also be useful for all antitumoral drugs that are substrates of P-pg with the advantage would be that of being able to reduce the dose of cytotoxic drugs by increasing their efficacy. Finally, the nanoliposomes described in this study have a protective value both for the molecules transported and for the healthy tissues in an in vivo administration. Toxicity of the nanoliposomes based on P90G and Chol is generally trivial, because of their composition. However, intravenously injected liposomes can interact with plasma proteins, and can stimulate or suppress the immune system due to their physical and chemical characteristics. Future studies will be oriented to the in vivo studies to examine the eventual intrinsic toxicity, the drugs’ retention, and circulation properties of the nanosystems, which are properties of great interest, due to an easy synthesis and scale-up by the pharmaceutical industry. 

## Figures and Tables

**Table 1:** Different combinations of lipids for the preparation of nanoliposomes.

P90G (mg)	P100H (mg)	Chol (mg)	PBS (mL)
330	/	10	10
330	/	20	10
330	/	100	10
660	/	100	10
660	/	200	10
650	/	300	10
/	330	10	10
/	330	20	10
/	330	100	10
/	500	10	10
/	500	20	10
/	500	100	10

**Table 2:** Different developed BRB-L.

P90G (mg)	Chol (mg)	BRB (mg)	PBS (mL)
330	10	7	10
330	20	7	10
330	100	7	10
660	100	7	10
660	200	7	10
650	300	7	10

**Table 3:** Physical characterization in terms of size, PdI, and ζ-potential of BRB-L. All results are expressed as mean ± SD (*n* = 3).

P90G/Chol Ratio (mg/mL)	Size (nm)	PdI	R (%)	EE (%)
33:1	138.3 ± 1.3	0.25 ± 0.01	/	/
33:2	154.9 ± 1.2	0.25 ± 0.01	/	31.18 ± 0.80
33:10	154.0 ± 1.6	0.20 ± 0.01	/	61.78 ± 0.40
66:10	127.8 ± 29.6	0.22 ± 0.03	103.41 ± 6.37	63.80 ± 6.41
66:20	162.6 ± 7.3	0.21 ± 0.02	98.92 ± 4.51	42.17 ± 6.49
65:30	230.3 ± 38.6	0.25 ± 0.07	92.36 ± 6.55	76.94 ± 8.04

**Table 4:** Physical and chemical characterization in terms of size, PdI, EE%, and R%, of the dialyzed nanoliposomes BRB-L and BRB/TAR-L at time zero (T_0_) and after 2 h incubation (T_2h_). All results are expressed as mean ± SD (*n* = 3).

Sample	Size (nm)	PdI	BRB R%	BRB EE%	TAR R%	TAR EE%
BRB-L T_0_	147.9 ± 3.7	0.219 ± 0.027	99.67 ± 0.41	64.15 ± 3.97	/	/
BRB-L T_2h_	150.6 ± 1.41	0.231 ± 0.004	/	60.66 ± 4.61	/	/
BRB/TAR-L T_0_	137.6 ± 0.6	0.276 ± 0.011	97.46 ± 1.46	73.83 ± 0.30	82.56 ± 0.38	76.44 ± 0.67
BRB/TAR-L T_2h_	135.3 ± 1.1	0.332 ± 0.022	/	71.36 ± 4.04	/	69.30 ± 5.81

## Supplementary Materials

The following are available online at https://www.mdpi.com/1999-4923/13/3/306/s1, Figure S1: K562 cells after 120-min incubated with peroxidase-containing liposomes. (A) The cells show prominent signs of degeneration and necrosis: the cell in the center still appears intact, while those at the margins show rupture of the plasma membrane and leakage of the cytoplasm. (B) Detail at higher magnification of the cell in A showing abnormal mitochondria with swollen cristae (arrows) and a plausible liposome within the cytoplasm (arrowhead). (C) Detail at higher magnification of the cell in A in which focal ruptures of the plasma membrane can be seen (demarcated by arrowheads). (D) Necrotic cells with extensive disruption of the cytoplasm and desegregation of chromatin; numerous liposomes can be seen close to plasma membrane remnants (arrow and insert); Figure S2: K562/DOXO cells after 120-min incubation with peroxidase-containing liposomes. (A) Representative images of cells showing prominent signs of degeneration and necrosis, i.e., marked mitochondrial swelling, clearing of the cytoplasmic and nuclear matrix with degenerated organelles and chromatin; two liposomes (insert) can be seen near the plasma membrane. (B) Detail of the cell in A showing swollen mitochondria with disrupted cristae (asterisks). (C) Cell undergoing necrosis, similar to that shown in (A); a liposome (insert) is visible near the plasmalemma. (D) Detail of the cell in (C) showing severe mitochondrial swelling, cytoplasmic vacuolation and rupture of the plasma membrane (demarcated by arrowheads); some vacuoles contain liposomes (insert and arrow).
