# Supplementary material for: Co-Delivery of Berberine Chloride and Tariquidar in Nanoliposomes Enhanced Intracellular Berberine Chloride in a Doxorubicin-Resistant K562 Cell Line Due to P-gp Overexpression
Source: Pharmaceutics. 2021 Feb 26;13(3):306. doi: 10.3390/pharmaceutics13030306 (PMC8025904; doi:10.3390/pharmaceutics13030306)
Supplement: Supplementary file 1 [file pharmaceutics-13-00306-s001.pdf]

pharmaceutics

MDPI

Supplementary Materials: Co-Delivery of Berberine Chloride and Tariquidar in Nanoliposomes Enhanced Intracellular Berberine Chloride in a Doxorubicin-Resistant K562 Cell Line Due to P-gp Overexpression

Giulia Vanti, Marcella Coronnello, Daniele Bani, Antonella Mannini, Maria Camilla Bergonzi, and Anna Rita Bilia

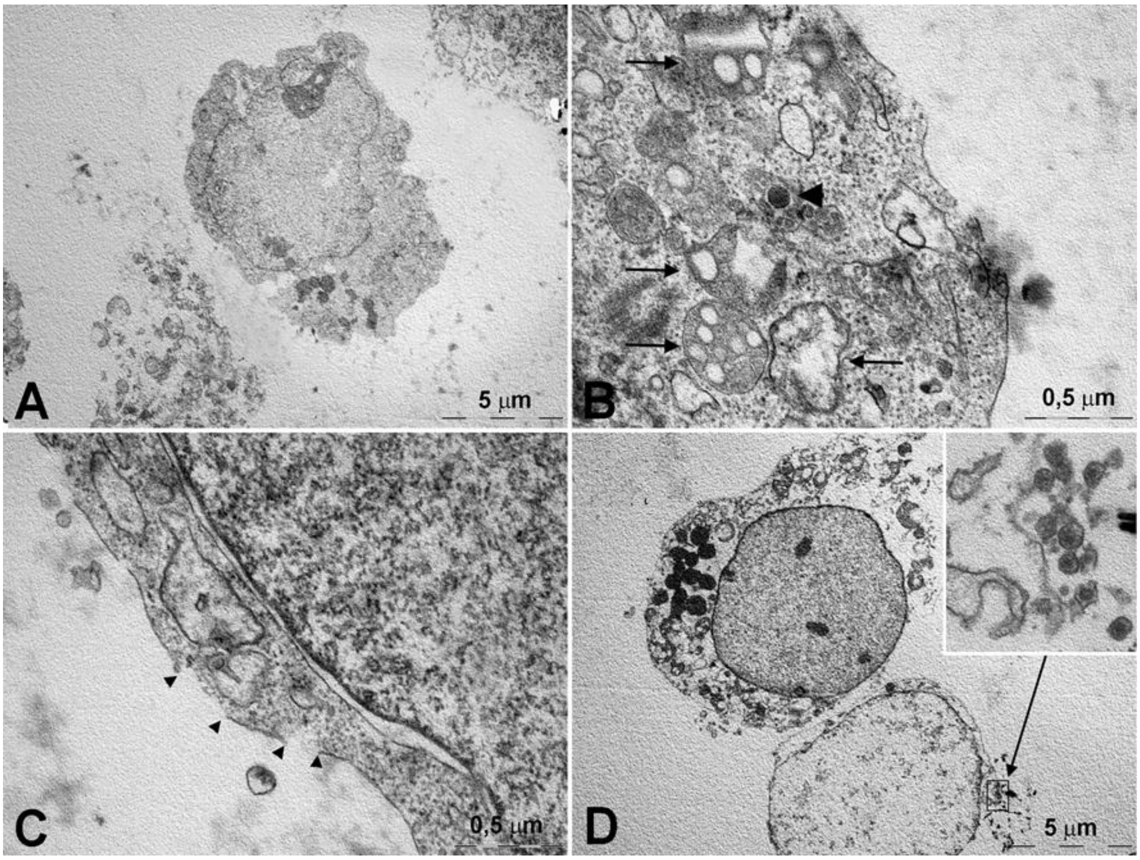

Figure S1: K562 cells after 120-min incubated with peroxidase-containing liposomes. (A) The cells show prominent signs of degeneration and necrosis: the cell in the center still appears intact, while those at the margins show rupture of the plasma membrane and leakage of the cytoplasm. (B) Detail at higher magnification of the cell in A showing abnormal mitochondria with swollen cristae (arrows) and a plausible liposome within the cytoplasm (arrowhead). (C) Detail at higher magnification of the cell in A in which focal ruptures of the plasma membrane can be seen (demarcated by arrowheads). (D) Necrotic cells with extensive disruption of the cytoplasm and disgregation of chromatin; numerous liposomes can be seen close to plasma membrane remnants (arrow and insert).

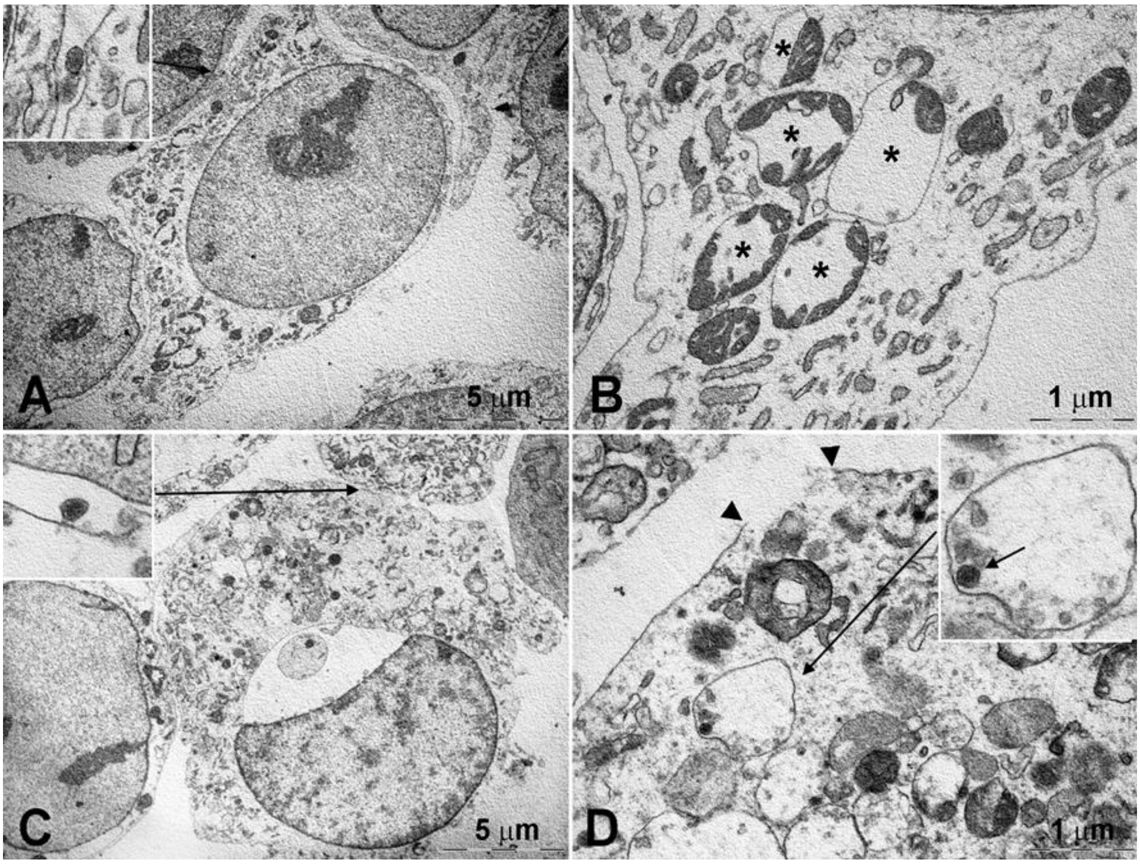

Figure S2: K562/DOXO cells after 120-min incubation with peroxidase-containing liposomes. (A) Representative images of cells showing prominent signs of degeneration and necrosis, i.e. marked mitochondrial swelling, clearing of the cytoplasmic and nuclear matrix with degenerated organelles and chromatin; two liposomes (insert) can be seen near the plasma membrane. (B) Detail of the cell in A showing swollen mitochondria with disrupted cristae (asterisks). (C) Cell undergoing necrosis, similar to that shown in (A); a liposome (insert) is visible near the plasmalemma. (D) Detail of the cell in (C) showing severe mitochondrial swelling, cytoplasmic vacuolation and rupture of the plasma membrane (demarcated by arrowheads); some vacuoles contain liposomes (insert and arrow).

Publisher's Note: MDPI stays neutral with regard to jurisdictional claims in published maps and institutional affiliations.

Copyright: © 2021 by the authors. Submitted for possible open access publication under the terms and conditions of the Creative Commons Attribution (CC BY) license (http://creativecommons.org/licenses/by/4.0/).
